# Effects of Heat Acclimation on Changes in Oxidative Stress and Inflammation Caused by Endurance Capacity Test in the Heat

**DOI:** 10.1155/2014/107137

**Published:** 2014-05-11

**Authors:** Triin Kaldur, Jaak Kals, Vahur Ööpik, Mihkel Zilmer, Kersti Zilmer, Jaan Eha, Eve Unt

**Affiliations:** ^1^Institute of Exercise Biology and Physiotherapy, University of Tartu, 18 Ülikooli Street, 50090 Tartu, Estonia; ^2^Estonian Centre of Behavioural and Health Sciences, University of Tartu, 18 Ülikooli Street, 50090 Tartu, Estonia; ^3^Institute of Biomedicine and Translational Medicine, University of Tartu, 19 Ravila Street, 50411 Tartu, Estonia; ^4^Department of Vascular Surgery, Tartu University Hospital, 8 Puusepa Street, 51014 Tartu, Estonia; ^5^Department of Cardiology, University of Tartu, 18 Ülikooli Street, 50090 Tartu, Estonia; ^6^Department of Sports Medicine and Rehabilitation, University of Tartu, 18 Ülikooli Street, 50090 Tartu, Estonia; ^7^Sports Medicine and Rehabilitation Clinic, Tartu University Hospital, 1a Puusepa Street, 50406 Tartu, Estonia

## Abstract

*Background*. The aim was to determine the effect of heat acclimation (HA) on oxidative stress (OxS) and inflammation in resting conditions and on the response pattern of these parameters to exhausting endurance exercise. * Methods*. Parameters of OxS and inflammation were measured in non-heat-acclimated status (NHAS) and after a 10-day HA program (i.e., in heat-acclimated status; HAS) both at baseline and after an endurance capacity (EC) test in the heat. * Results*. As a result of HA, EC increased from 88.62 ± 27.51 to 161.95 ± 47.80 minutes (*P* < 0.001). HA increased OxS level: total peroxide concentration rose from 219.38 ± 105.18 to 272.57 ± 133.39 **μ**mol/L (*P* < 0.05) and oxidative stress index (OSI) from 14.97 ± 8.24 to 20.46 ± 11.13% (*P* < 0.05). In NHAS, the EC test increased OxS level: total peroxide concentration rose from 219.38 ± 105.18 to 278.51 ± 125.76 **μ**mol/L (*P* < 0.001) and OSI from 14.97 ± 8.24 to 19.31 ± 9.37% (*P* < 0.01). However, in HAS, the EC test reduced OSI from 20.46 ± 11.13 to 16.83 ± 8.89% (*P* < 0.05). The value of log high-sensitive C-reactive protein increased from −0.32 ± 0.32 to −0.12 ± 0.34 mg/L (*P* < 0.05) in NHAS and from −0.31 ± 0.47 to 0.28 ± 0.46 mg/L (*P* < 0.001) in HAS. * Conclusion*. HA increases OxS level. However, beneficial adaptive effects of HA on acute exhaustive exercise-induced changes in OxS and inflammation parameters occur in a hot environment.

## 1. Introduction

Increased oxidative stress (OxS) is considered to be associated with both aerobic and anaerobic [1] exercise in young and old individuals [2]. OxS has also been demonstrated to be dependent on both intensity and duration of physical activity [3–6]. There is evidence that OxS is lower in physically fit and active adults compared with less fit or sedentary individuals [7].

Results of animal studies suggest that acute exposure to high temperatures may result in increased OxS [8] and OxS should be considered as part of the stress response to heat exposure [9]. Mills et al. [10] showed an increase in OxS in hyperthermic horses, who were used as a model of an elite athlete exercising to fatigue. In addition, OxS was exacerbated during exercise in high temperature environment. Investigations in humans also reveal that high ambient temperature and hyperthermia may increase exercise-induced OxS response [11, 12]. On the contrary, systematic endurance training may alleviate OxS and elevate antioxidant status following exhausting exercise [13, 14]. Physical activity has been shown to have an effect on inflammatory markers [15]. In the acute phase response to strenuous exercise, inflammation is increased for a short term [16, 17], but this transient acute phase response may be reduced by exercise training [18, 19].

To our knowledge, there are very few reports regarding the impact of combined heat and exercise stress occurring during prolonged exercise in the heat on parameters of OxS and inflammation. We have recently demonstrated that heat acclimation (HA) improves arterial elasticity [20], which has been shown to be directly linked to OxS [21] and inflammation [22]. However, there are no published reports about the impact of HA on OxS and inflammation induced by exhaustive exercise in the heat. Therefore, the aim of this study was to determine the effect of HA on the parameters of OxS and inflammation in resting conditions and on the response pattern of these parameters to exhausting endurance exercise in the heat.

## 2. Materials and Methods

### 2.1. Ethical Approval

The study was approved by the Research Ethics Committee of the University of Tartu. The study conformed to the principles outlined in the Helsinki Declaration, and all subjects provided their written informed consent before inclusion in the study.

### 2.2. Subjects and Study Design

Twenty-one physically active young men participated in this study (age: 24.9 ± 3.7 yrs; height 1.83 ± 0.06 m; weight 80.3 ± 9.4 kg; body mass index: 24.16 ± 2.73;* V*O_2_ peak 53.8 ± 7.1 mL/kg/min; heart rate 56.36 ± 9.35 beats/min; systolic blood pressure 122.14 ± 9.65 mmHg; diastolic blood pressure 65.88 ± 7.52 mmHg). None of the participants were taking any medications, and none were smoking or had a history of heat illness. To ensure standardization of nutritional status prior to the study, the subjects were instructed to follow healthy diet, to keep the diet stabilized, and to avoid any use of additional food supplements 2 months prior to participation in study.

The current investigation was a constituent part of a complex HA study [20, 23, 24]. To avoid natural HA, the study was conducted during the winter-spring period in Estonia. Parameters of OxS and inflammation were measured 4 times: 2 times at non-heat-acclimated status (NHAS) (at baseline and after endurance capacity (EC) test in the heat) and 2 times at heat-acclimated status (HAS) (at baseline and after EC test in the heat).

### 2.3. Measurement of Parameters of Oxidative Stress and Inflammation

All blood measurements were performed at the Institute of Biomedicine and Translational Medicine (University of Tartu) and Laboratory Department of Tartu University Hospital. All determination procedures were performed in accordance with the manufacturer's recommendation. Venous blood samples were obtained from the antecubital fossa between 8:00 and 10:00 following an overnight fast. In order to ensure standardization of physiological parameters, the blood samples were obtained after at least 1 hour of rest in supine position [20]. White blood cells (WBC) counts, hemoglobin, and hematocrit were assessed in whole blood using a QBC Autoread Plus autoanalyzer (QBC Diagnostics, Inc., USA). Other blood samples were centrifuged within 15 minutes after collection at 3000 rpm for 15 minutes to obtain plasma or serum. All the plasma/serum samples were stored at −70°C until the analysis.

The plasma high-sensitive C-reactive protein (hsCRP) was measured by a validated latex particle-enhanced high-sensitivity immunoturbidimetric assay (CRP Latex HS, Roche Diagnostics Gmbh, Mannheim, Germany) and analyzed by the Hitaci 912 analyzer (Roche Diagnostics, Basel, Switzerland). Total peroxide concentrations of samples were determined using OXYSTAT Assay Kit catalogue number BI-5007 (Biomedica Gruppe, Biomedica Medizinprodukte GmbH & Co Kg, Wien). Total antioxidant capacity (TAC) was measured by using an automated measurement method by Erel [25]. Percent ratio of the total peroxide concentration of plasma to the TAC of plasma was accepted as oxidative stress index (OSI), which is an indicator of the degree of OxS. Commercially available enzyme-linked immunosorbent assay kits were used to determine serum oxidized low-density lipoproteins (oxLDL) (Mercodia AB, Uppsala Sweden; catalogue number 10-1143-01) and serum soluble intercellular adhesion molecule-1 (sICAM-1) (Human soluble ICAM-1 Immunoassay, catalogue number BBE 1B, R&D Systems Inc., Minneapolis, USA). Plasma *β*_2_-microglobulin (*β*_2_ M) concentration was measured by a chemiluminescent immunoassay using a commercially available kit (L2KBM2, Siemens Medical Solutions Diagnostics, California, USA) in the IMMULITE 2000 automated analyzer (Siemens Medical Solutions Diagnostics, California, USA). The Evidence Investigator Cytokine and Growth Factors High-Sensitivity Array based on the sandwich chemiluminescent immunoassay (Randox Laboratories Ltd CTK HS catalogue number EV 3623) was used for simultaneous quantitative detection of multiple related cytokines: interleukin-6 (IL-6), interleukin-8 (IL-8), epidermal growth factor (EGF), vascular endothelial growth factor (VEGF), and monocyte chemoattractant protein-1 (MCP-1) from a single patient sample. The core technology is the Randox Biochip containing an array of discrete test regions of immobilized antibodies specific to different cytokines and growth factors. N-terminal probrain natriuretic peptide (NT-proBNP) was measured using electrochemiluminescence immunoassay ECLIA and an Elecsys 2010 analyzer (Roche Diagnostics). Relative changes in plasma volume after the endurance capacity test in NHAS and HAS were calculated on the basis of hemoglobin and hematocrit values [26].

### 2.4. Measurement of Peak Oxygen Uptake

To establish the workload for the protocol of the EC test and HA program, each subject was initially tested for peak oxygen uptake (*V*O_2_ peak) as described by Burk et al. [23]. The test was conducted in thermoneutral conditions (20–22°C) on a motorized treadmill (Viasys/Jaeger LE300 C, Viasys Healthcare GmbH, Hoechberg, Germany) using an online breath-by-breath metabolic system (MasterScreen CPX, Viasys Healthcare GmbH, Hoechberg, Germany).* V*O_2_ peak was considered to be the mean of the three highest consecutive 15 s recordings at the end of the test. Secondary criteria for achieving* V*O_2_ peak included respiratory exchange ratio > 1.15 and heart rate > 95% of the subject's age-predicted maximum.

### 2.5. Endurance Capacity Test in the Heat

**Figure 1:**
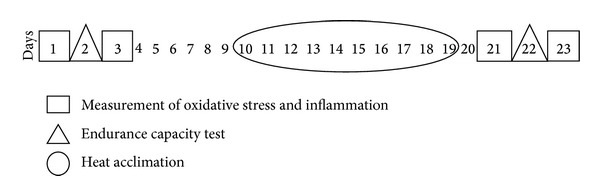
Study design.

EC test in the heat was performed twice, before and after the HA program (Figure 1), on a treadmill in a climatic chamber (Design Environmental Ltd., Gwent, South Wales, UK) in the heat (42°C; relative humidity 18%). To control the status of hydration of our subjects on the days of the endurance capacity test, the subjects voided and donated a urine sample for specific gravity measurement, which was performed by means of a digital clinical refractometer (PDX-CL, VeeGee Scientific Inc., Kirkland, WA, USA). Their body mass was also measured using an electronic scale (CH3G-150I Combics, Sartorius AG, Goettingen, Germany) to the nearest 0.001 kg before and after the EC test. The intensity of work during the test was adjusted to 60% of the subject's personal* V*O_2_ peak and was controlled by changing the grade of the treadmill belt in the range of 7–15%, whereas speed was kept constant at 6 km h^−1^. The test was performed until exhaustion or until indications for the termination of the test occurred (core temperature above 40°C for more than 5 minutes; heart rate above 95% of the subject's age-predicted maximal heart rate for at least 5 minutes; occurrence of symptoms of exertional heat illness, such as nausea, headaches, and dizziness).

### 2.6. Heat Acclimation Program

The HA program employed in the current study lasted 10 consecutive days and was a modification of that previously used by Yamada et al. [27]. The subjects exercised daily in a climatic chamber maintained at a hot temperature (42°C; relative humidity 18%) for 110 minutes (two 50 min bouts of exercise with 10 min of rest between bouts). The intensity of exercise was 55% of* V*O_2_ peak during the first 5 days and the workload was raised to the level of 60% of* V*O_2_ peak for the second 5 days of the HA protocol. The constant speed of 6 km h^−1^ was employed and the grade of the belt of the treadmill was regulated in the range of 5–15%.

To detect clinical symptoms of heat illness, core temperature* via *a rectal probe (TX-2, Columbus Instruments, Columbus, OH, USA) and heart rate* via *a transmitter strap (Suunto Dual Belt, Suunto OY, Finland) were monitored continuously throughout all heat exposure. Termination criteria for daily exposure included the following: (1) completion of the protocol; (2) a subject's request to stop; (3) a rise of core temperature to 39.5°C for 5 min; (4) a rise of heart rate to 95% of maximal heart rate for 5 min; (5) symptoms of exertional heat illness.

### 2.7. Data Analysis

The Statistical Package for the Social Sciences (SPSS, version 20.0) software was used to perform all tests. Continuous variables are shown as a mean ± standard deviation (*x* ± SD). The Kolmogorov-Smirnov test was used to check all data for normal distribution. Because of the skewed distribution of hsCRP, logarithmic transformation was performed before statistical analysis. The effect of acclimation was analyzed with repeated measures analysis of variance. Bonferroni post hoc analysis was used to evaluate the differences in values of parameters in heat-acclimated/non-heat-acclimated status before and after EC test. A Pearson and Spearman product moment coefficient of correlation was used to determine the relationships among variables of oxidative stress and inflammation. For all statistical analyses, the 0.05 level of significance was used.

## 3. Results 

**Figure 2:**
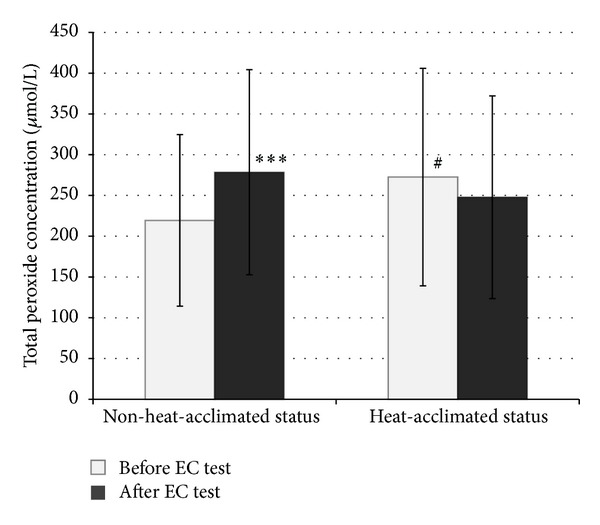
The values of total peroxide concentration before and after the endurance capacity test in the heat measured before and after heat acclimation program (*x* ± SD). EC: endurance capacity; ***P < 0.001 as compared to value measured before EC test; ^#^ *P* < 0.05 as compared to non-heat-acclimated status before EC test; main effect (*F*): 3.79; *P* value: 0.035.

**Figure 3:**
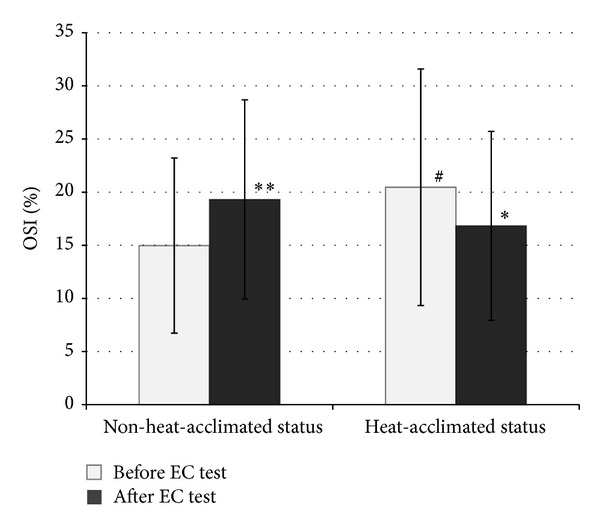
The values of oxidative stress index before and after the endurance capacity test in the heat measured before and after heat acclimation program (*x* ± SD). EC: endurance capacity; OSI: oxidative stress index; *P < 0.05; **P < 0.01 as compared to value measured before EC test in heat-acclimated status/non-heat-acclimated status; ^#^ *P* < 0.05 as compared to non-heat-acclimated status before EC test; main effect (*F*): 3.79; *P* value: 0.015.

**Figure 4:**
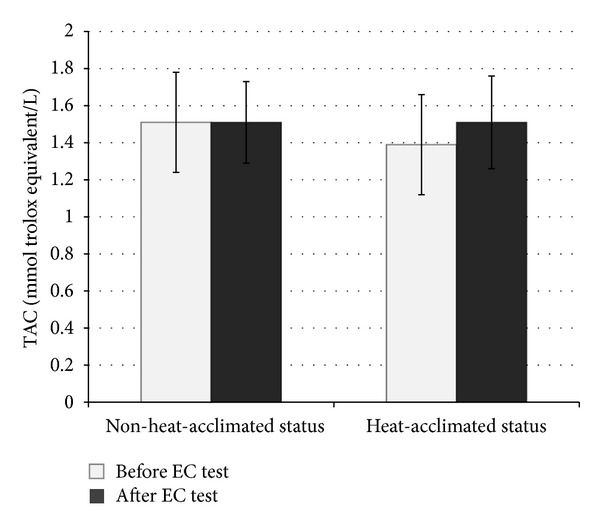
The values of total antioxidant capacity before and after the endurance capacity test in the heat measured before and after heat acclimation program (*x* ± SD). EC: endurance capacity; TAC: total antioxidant capacity; main effect: (*F*) 1.39; *P* value: 0.254.

**Figure 5:**
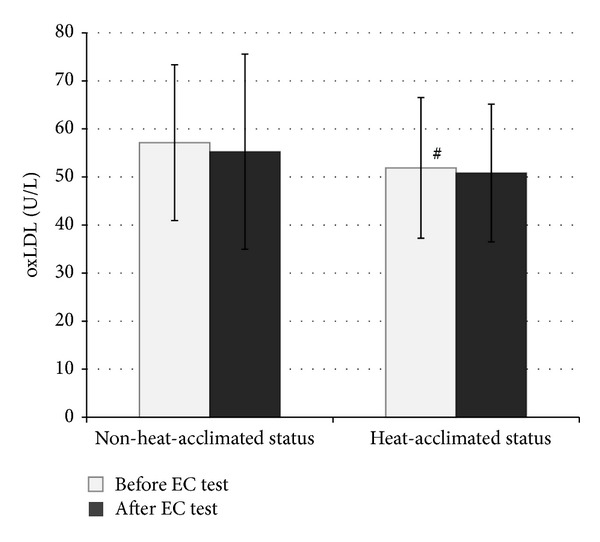
The values of oxidized low-density lipoproteins before and after the endurance capacity test in the heat measured before and after heat acclimation program (*x* ± SD). EC: endurance capacity; oxLDL: oxidized low-density lipoproteins; ^#^ *P* < 0.05 as compared to non-heat-acclimated status before EC test; main effect (*F*): 2.83; *P* value: 0.046.

Similar preexercise urine specific gravity and body mass values reveal that our subjects were in a similar hydration status at the beginning of the EC test (at heat-acclimated status and non-heat-acclimated status). After the HA program, all subjects showed improvement in EC: their mean EC increased from 88.62 ± 27.51 to 161.95 ± 47.80 minutes (*P* < 0.001). The values of OxS parameters measured before and after the EC test in the heat in both NHAS and HAS are shown in Figures 2, 3, 4, and 5. 10-day HA program increased (*P* < 0.05) total peroxide concentration (24.2%) and OSI (36.7%) and decreased oxLDL (9.2%) but had no impact on TAC (*P* > 0.05), measured at NHAS and HAS before EC test (Figure 1). The EC test performed at NHAS increased total peroxide concentration (27%; *P* < 0.001) and OSI (29%; *P* < 0.01), whereas TAC and oxLDL remained unchanged (*P* > 0.05). At HAS, the EC test decreased OSI (17.7%; *P* < 0.05) but had no impact (*P* > 0.05) on total peroxide concentration, TAC, or oxLDL. The 10-day HA program had no impact on inflammation parameters measured in this study (*P* > 0.05) (Table 1), measured at NHAS and HAS before EC test. The EC test increased significantly hsCRP and log hsCRP in both NHAS and HAS. There was no impact (*P* > 0.05) of EC test in any other inflammation parameter in NHAS. However, in HAS, the EC test decreased significantly the concentrations of MCP-1 (*P* < 0.001). In addition, the EC test increased the NT-proBNP level from 19.57 ± 10.43 to 28.79 ± 15.83 pg/mL in NHAS (*P* < 0.05) but had no significant impact on this parameter in HAS (27.90 ± 18.15 and 30.90 ± 15.55 pg/mL, before and after EC test, resp.; *P* > 0.05) (main effect (F) 2.98; *P* value 0.038).

Our statistical analysis did not reveal any statistically significant correlations between oxidative and inflammation parameters.

## 4. Discussion

The main novel finding of this study is that 10-day HA program increases OxS level but induces beneficial adaptive effects on the responses of OxS and inflammation parameters to acute exhausting endurance exercise in the heat in young healthy men.

Specifically, we observed 36.7% (*P* < 0.05) higher OSI level before EC test condition in our subjects in HAS compared to NHAS (Figure 3). OSI reflects both oxidative and antioxidative factors and is considered a reliable marker of OxS [28]. The increase in OSI was mainly caused by elevated (24.2%; *P* < 0.05) total peroxide concentration (Figure 2).

There is no earlier compelling evidence about the effect of HA program on oxidative stress parameters. In our study, the changes in OxS parameters were induced by the combined effect of the concurrence of exercise and heat stress. Goto and colleagues [29] showed increased indices of oxidative stress after high-intensity exercise training for 12 weeks in healthy young men. Exercise-induced physiological strain is also increased by hyperthermia [30]. Hyperthermia depends on the environmental temperature in which the exercise is performed and, according to* in vitro* evidence by Lin et al. [9], the OxS is considered one part of the stress response to heat exposure. It is suggested that core body temperature during exercise is a factor participating in the induction of OxS [31]. In addition, the study by Quindry et al. [12] found that moderate intensity exercise in a warm environment elicits a blood OxS response and indicated that exercise-induced OxS can be influenced by environmental temperature.

In addition to the increased level of OxS after the 10-day HA, we also observed an increase in OxS level after acute exhausting endurance exercise in the heat. The EC test in young healthy men in NHAS induced a mean increase in total peroxide concentration up to 27% (*P* < 0.001) and an increase in mean value of OSI (29%; *P* < 0.01) (Figures 2 and 3). Our finding is in agreement with the studies by Demirbag et al. [32] and Aguiló et al. [33], where an increased OxS level was found. The shift towards OxS in the balance between OxS and antioxidant status after short-term exercise is also demonstrated by Kurkcu [34].

Furthermore, our study revealed that HA induces beneficial adaptive effects on the responses of OxS and inflammation parameters to acute exhausting endurance exercise in the heat. When the EC test was performed in NHAS, the OxS parameters increased significantly after the EC test (e.g., an increase in total peroxide concentration and OSI). However, when the EC test was performed in HAS, the OSI decreased 17.7% (*P* < 0.05) as compared to the value measured before the EC test (Figure 3). We suggest that the observed beneficial adaptive effect of HA on the OxS level caused by the EC test can at least partly be due to heat shock proteins (HSP). HSP may be important modifying factors in cellular responses to a variety of physiologically relevant conditions such as hyperthermia, exercise, oxidative stress, and metabolic challenge and modifying factors in acquired thermotolerance [35]. It is well known that heat and exercise greatly accelerate the synthesis of the inducible HSP (especially Hsp70) [36, 37], which is thought to have both a cellular and systemic protective role [35]. Basal levels of serum Hsp70 increased significantly over the 15 days of HA and the increase in Hsp70 after exercise was inversely correlated to the resting values of Hsp70 [38].

In addition to the reduction of OxS parameters after the EC test in HAS, our results revealed significantly decreased value of inflammatory marker MCP-1 (26.3%; Table 1). In our subjects, the average log hsCRP increased significantly after the EC test at NHAS as well as at HAS as compared to before EC test values. The highest value of log hsCRP was detected after the EC test at HAS. However, the absolute values of hsCRP were relatively low (mean value less than 4 mg/L) in our study. A study by Weight and colleagues [16] reported an acute rise in hsCRP after the strenuous long-lasting exercise up to 2000%; hsCRP values returned to baseline two to six days after exercise. Our previous study [39] showed a 57% increase in hsCRP 24 hours after three days of extreme physical load in well-trained cadets, whereas the increase of hsCRP was inversely related to subjects'* V*O_2_ max level. Furthermore, a high CRP level is considered a significant independent risk factor for coronary heart disease [40]. It is understood that the components of immune function are affected by physical activity in an adverse environment and that endurance exercise may be capable of inducing a subclinical pyrogenic response [41]. During prolonged exercise and/or heat stress, the level of inflammatory cytokines increases and heat exposure tends to stimulate the release of IL-6 [42], which can raise the thermoregulatory set point resulting in increased heat storage. It is known that the short-term transient increase in hsCRP after exercise is mediated by the cytokine system and mainly by IL-6 [15]. Regular exercise training and a good fitness level [15, 39] may alleviate this response. In our study the value of IL-6 increased 17.9% (*P* > 0.05) in NHAS and decreased 18.9% (*P* > 0.05) in HAS after the EC test in the heat.

In the present study, evoked changes were also detected in growth factors: after the EC test at HAS the mean decrease in EGF was 25.7% and in the case of VEGF was 16.4% (*P* > 0.05). We suggest that decreases in these angiogenesis promoters may be at least in part the effect of improved vascular elasticity [20], which may reduce the total cardiovascular burden. Our study results reveal that the cardiovascular strain was smaller after the EC test at the HAS, as the EC test increased NT-proBNP value at NHAS, but not at HAS. These results are in agreement with our previous findings [20] that our subjects were acclimated through a 10-day exhaustive exercise program in the heat. As discussed earlier [20, 23] and in agreement with the findings of several studies [27, 43, 44], after the HA program, all our subjects showed improvement in EC, where the subjects' mean EC increased 86%.

Our study has some limitations. Firstly, we are unable to measure the role of antioxidative components in changes in oxidative stress and inflammation parameters. However, the subjects were instructed to keep their diet stabilized and to avoid any use of additional food supplements 2 months prior to participation in the study. Secondly, we did not measure the role of exercise-induced mechanical damage to muscle fibers in the changes in the measured parameters. The possibility that to some extent the changes in oxidative stress and inflammation parameters were induced by mechanical damage cannot be excluded, but taking into account the moderate intensity of exercise, we do not consider this likely. Thirdly, from the methodological point of view, the existence of a control group would have been appropriate to distinguish the effect of heat stress alone but as our investigation was a part of a complex HA study, the existence of a control group was too complicated to achieve.

The findings of our study have significant implications for future research. As the sample size was small, the results of our study should be verified in large-scale investigations. Future trials could focus on different age and gender groups as well as on participants with different fitness levels. Further research is also warranted in order to assess whether those HA effects could be caused by exercise or heat stress alone. Preclinical studies are needed to understand the exact mechanism by which HA has the effects on changes in oxidative stress and inflammation caused by an endurance capacity test in the heat.

In conclusion, the present study demonstrates that a 10-day HA program statistically significantly increases the OxS level. In addition, HA induces beneficial adaptive effects on the responses of OxS and inflammation parameters to acute exhausting endurance exercise in the heat in young healthy men. The preliminary findings of this study have significant clinical implications in the monitoring of adaptation to HA, which is important for individuals, who have to be physically active in thermally stressful environmental conditions (e.g., soldiers and athletes). Our results show that several beneficial adaptive effects of HA on exhaustive exercise-induced changes in OxS and inflammation parameters in the heat could successfully be achieved within a short period of time.

## Figures and Tables

**Table 1:** The values of inflammation parameters before and after the endurance capacity test in the heat measured before and after heat acclimation program (*x*  ± SD).

Parameters of inflammation	Non-heat-acclimated status		Heat-acclimated status		Main effect (*F*)	*P* value
	Before EC test	After EC test	Before EC test	After EC test		
hsCRP mg/L	0.63 ± 0.52	1.04 ± 0.89*	1.17 ± 2.59	3.53 ± 4.64*	5.97	0.010

log⁡hsCRP mg/L	−0.32 ± 0.32	− 0.12 ± 0.34*	− 0.31 ± 0.47	0.28 ± 0.46***	14.76	0.000

sICAM-1 ng/mL	163.62 ± 29.62	169.20 ± 30.79	164.48 ± 28.17	169.22 ± 27.31	0.95	0.402

WBC ×10^9^/L	5.33 ± 1.13	5.68 ± 1.42	5.30 ± 1.08	5.72 ± 1.22	1.72	0.173

EGF pg/mL	2.81 ± 1.99	3.19 ± 2.26	3.31 ± 2.33	2.46 ± 1.77	2.16	0.124

VEGF pg/mL	53.14 ± 45.04	54.99 ± 36.04	54.87 ± 35.89	45.87 ± 29.38	1.16	0.325

MCP-1 protein-1 pg/mL	157.52 ± 76.74	148.64 ± 60.20	187.79 ± 76.37	138.36 ± 58.05***	6.10	0.001

IL-6 pg/mL	0.78 ± 0.67	0.92 ± 0.67	1.11 ± 1.51	0.90 ± 0.93	0.93	0.433

IL-8 pg/mL	8.55 ± 12.25	9.93 ± 17.48	9.92 ± 13.05	8.30 ± 12.63	2.11	0.152

*β*2M *μ*g/L	1478.29 ± 217.79	1465.42 ± 204.64	1453.24 ± 213.02	1528.18 ± 280.28	1.73	0.538

Note: EC: endurance capacity; hsCRP: high-sensitive C-reactive protein; sICAM-1: soluble intercellular adhesion molecule-1; WBC: white blood cells; EGF: epidermal growth factor; VEGF: vascular endothelial growth factor; MCP-1: monocyte chemoattractant protein-1; IL-6: interleukin-6; IL-8: interleukin-8; *β*2M: *β*_2_-microglobulin;  *P < 0.05; ***P < 0.001 as compared to value measured before EC test.
